# Co-Occurrence of Metal Contaminants in United States Public Water Systems in 2013–2015

**DOI:** 10.3390/ijerph18157884

**Published:** 2021-07-26

**Authors:** Alesha K. Thompson, Michele M. Monti, Matthew O. Gribble

**Affiliations:** 1 Gangarosa Department of Environmental Health, Rollins School of Public Health, Emory University, 1518 Clifton Road NE, Atlanta, GA 30322, USA; matt.gribble@emory.edu; 2 National Center for Environmental Health (NCEH), Centers for Disease Control and Prevention, 4770 Buford Highway, Atlanta, GA 30341, USA; mmonti@cdc.gov

**Keywords:** environmental monitoring, co-occurrence, environmental exposure

## Abstract

The United States Environmental Protection Agency monitors contaminants in drinking water and consolidates these results in the National Contaminant Occurrence Database. Our objective was to assess the co-occurrence of metal contaminants (total chromium, hexavalent chromium, molybdenum, vanadium, cobalt, and strontium) over the years 2013–2015. We used multilevel Tobit regression models with state and water system-level random intercepts to predict the geometric mean of each contaminant occurring in each public water system, and estimated the pairwise correlations of predicted water system-specific geometric means across contaminants. We found that the geometric means of vanadium and total chromium were positively correlated both in large public water systems (r = 0.45, *p* < 0.01) and in small public water systems (r = 0.47, *p* < 0.01). Further research may address the cumulative human health impacts of ingesting more than one contaminant in drinking water.

## 1. Introduction

There are several heavy metals found in United States drinking water that are naturally occurring and, although human activities can increase the concentrations available, fully eliminating these exposures from the environment is impossible [1,2,3]. The Environmental Protection Agency (EPA) currently only regulates ten heavy metals in public water systems, while numerous metals remain unregulated, including cobalt, molybdenum, strontium, and vanadium [4,5,6,7,8]. Research has shown that some heavy metals may cause negative health effects, such as cancers and kidney, liver, and neurological damage [9,10]. Understanding which metals pose health risks when ingested, and at what doses, is a priority for environmental health practitioners. Human populations are exposed to a mixture of metals with uncertain implications for human health [9,10].

The United States has a surveillance system in place for these contaminants and they store multiple years of data in the National Contaminant Occurrence Database [11]. The EPA monitors unregulated contaminants in drinking water through the Unregulated Contaminant Monitoring Rule (UCMR) [4,5,6,7,8]. The Contaminant Candidate List (CCL) is part of a decision-making process that informs each iteration of the UCMR [8]. Through a multi-year process, organic contaminants, hormones, synthetic compounds, and heavy metals are selected on criteria set forth by the Safe Drinking Water Act for evaluation under the CCL [4,5,6,7,8]. Contaminants from the CCL are then chosen for monitoring under the UCMR if they satisfy two conditions: first, the contaminant has potential to cause health effects and, second, the contaminant occurs in water systems at concentrations that would constitute a public health concern [4,5,6,7,8]. This is a 3–4-year process, wherein selected public water systems are regularly sampled for the contemporary list of UCMR contaminants [4,5,6,7,8].

The objective of this secondary data analysis of heavy metal measurements collected for the third wave of the EPA’s UCMR process (UCMR 3) was to assess the co-occurrence of contaminants salient to cumulative risk assessment of these chemicals. 

## 2. Materials and Methods

The Environmental Protection Agency UCMR 3 dataset contained detailed information about each sample obtained by each water system in the National Contaminant Occurrence Database on the US EPA website [11,12]. The third wave of UCMR monitoring took place from January 2013 to December 2015 and all water systems serving over 10,000 people (large systems) were required to monitor for the List 1 contaminants. Eight hundred water systems that served no more than 10,000 people (small systems) were randomly selected to also monitor for the contaminants. Several heavy metals (total chromium (Cr), hexavalent chromium (Cr (VI)), strontium (Sr), molybdenum (Mo), vanadium (V), and cobalt (Co)) were included on the UCMR 3 assessment list. Total chromium was monitored due to the requirements under the Safe Drinking Water Act and included all forms of chromium, including hexavalent chromium [13]. Public water systems were required to sample groundwater and surface water. Systems were to sample groundwater twice in one consecutive 12-month period and these samples were obtained from 5 to 7 months apart. Surface water was sampled in 4 consecutive quarters and samples were obtained 3 months apart. The water was sampled at the entry point to the distribution system as well as distribution system maximum-residence time-sampling locations, resulting in over 377,000 water samples for these heavy metals, collectively [14,15,16,17]. Samples were then distributed to EPA-approved laboratories, and results of the monitoring were stored in the National Drinking Water Contaminant Occurrence Database [14,15,16,17]. The final dataset included 4,123 large water systems (serving >10,000 people) and 799 small water systems. There were different total numbers of public water systems in the dataset for different contaminants (Table 1). 

In the database, the Public Water System ID and Public Water System Name variables identify each water system that sampled for the contaminants. The database also contains an indicator to identify each sample as above or below the Minimum Reporting Level (MRL) [14,15,16,17]. The MRL varies by contaminant based on the minimum detectable concentration for the analytical method associated with each contaminant [14,15,16,17]. The number of water systems that reported all concentration values below the MRL is reported in Table 1.

Tobit regression models account for censored data [18,19,20,21] and mixed-effect Tobit models further account for clustering of observations in a dataset [21]. Mixed-effect Tobit regression models of the log-transformed contaminants, left-censored at the log-transformed MRL, with normally distributed random intercepts for public water system and state, but no fixed effects, were used to predict the mean log-concentrations of each contaminant for each public water system. Models were fitted for each contaminant separately. The predicted mean log-concentrations were exponentiated to obtain geometric means of each contaminant predicted for each water system. If a water system reported all concentration values below the MRL, the model predicted the value based on other public water systems within the state and the average level across states. To assess co-occurrence of contaminants in public water systems, the predicted system-level predicted geometric means were compared pairwise across chemicals. A Pearson correlation [22] of the predicted geometric means was used to assess strength of the relationships between chemicals in public water systems. Correlations were considered Bonferroni-significant if *p* < 0.0033 [23]. The models were fitted separately to large public water systems and small water systems. 

All mixed-effect Tobit regression models were implemented using Stata 15.1 S/E.

## 3. Results

**Figure 1:**
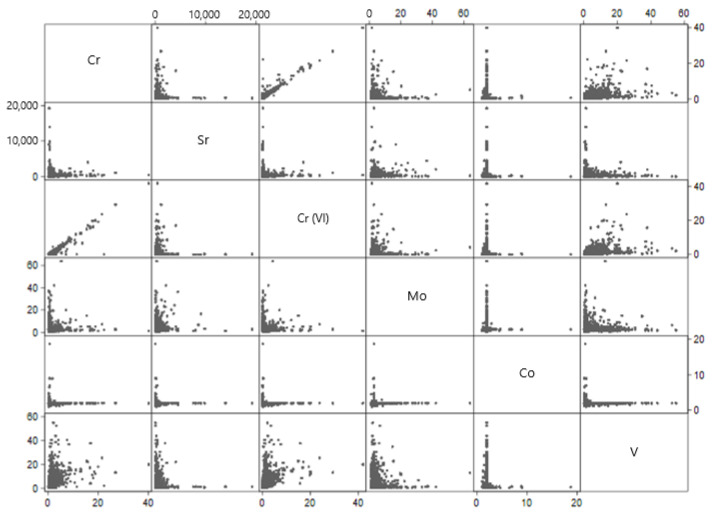
Predicted large public water system-level geometric means of contaminants. Large water system-level geometric means were obtained from mixed-effect Tobit regression models fitted to each contaminant separately, with two random intercepts accounting for nesting of water samples within water systems within states. Each axis corresponds to the contaminants’ range of geometric means (µg/L).

There were several relationships between chemical contaminants in large public water systems (Table 2, Figure 1). There was a high correlation between total chromium and hexavalent chromium in public water systems (r = 0.984, *p* < 0.01). There was a noteworthy positive correlation between vanadium and hexavalent chromium in public water systems (r = 0.445, *p* < 0.01), as well as between vanadium and total chromium (r = 0.448, *p* < 0.01). 

Cobalt was generally anticorrelated with other metals in large water systems. As cobalt increased, chromium (r = −0.017, *p* < 0.01), strontium (r = −0.024, *p* < 0.01), hexavalent chromium (r = −0.021, *p* < 0.01) and vanadium (r = −0.046, *p* < 0.01) decreased.

**Figure 2:**
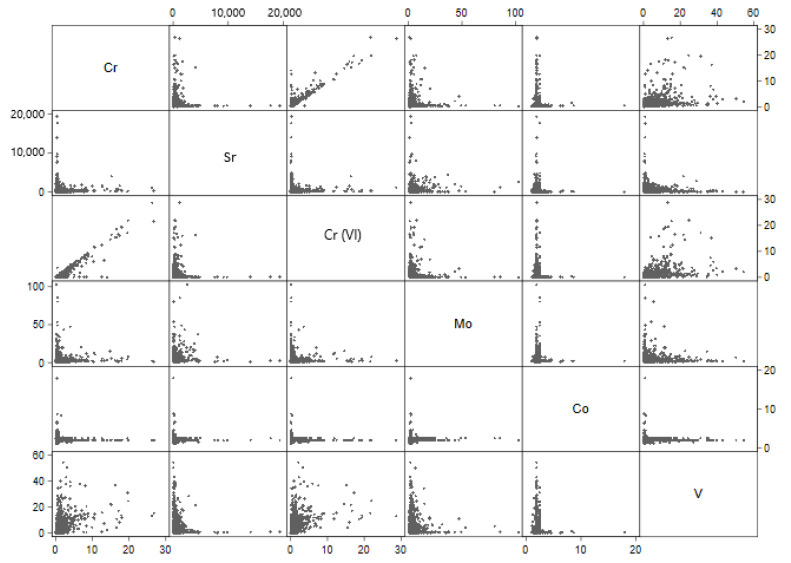
Predicted small public water system-level geometric means of contaminants. Small water system-level predicted geometric means were obtained from mixed-effect Tobit regression models fitted to each contaminant separately, with two random intercepts accounting for nesting of water samples within water systems within states. Each axis corresponds to the contaminants’ range of predicted geometric means (µg/L).

Among small water systems serving ≤10,000 people there were similar patterns of association (Table 3, Figure 2). The most noteworthy positive correlations were between total chromium and vanadium (r = 0.474, *p* = < 0.01) and between hexavalent chromium and vanadium (r = 0.498, *p* = < 0.01).

## 4. Discussion

This paper presents an assessment of co-occurring metals in the United States public water systems. To accommodate values below the MRL, we made an inference about the co-occurrence in drinking water systems based on aggregated data summaries (i.e., predicted geometric means of contaminants in each water system). Our finding is therefore vulnerable to potential ecological fallacy in conclusions about contaminant co-occurrence in individual water samples [24], and if the contaminants have short biological half-lives in humans from drinking water [25,26], for toxicologically relevant joint doses of these chemicals. Nevertheless, our finding that vanadium and chromium tend to both be high in the same water systems raises concerns about potential impacts of joint exposure warranting a cumulative risk assessment approach [27,28] and further targeted investigation into possible human joint exposures. We think our approach resulted in a conservative estimate of the correlations between these six metals at the water system level, as we predicted the geometric means for each chemical separately rather than jointly modeling these contaminants in a multivariate outcome model.

There are additional potential sources of bias in these estimates. It is possible that differences in contaminant levels between water systems could have been confounded by seasonality. The samples collected by the EPA were collected a minimum of 3 or 6 months apart, depending on the type of water, but not necessarily at the same dates in different locations. If rainfall were heavier in certain months, there could have been changes in contaminant hydrology affecting the estimated means [29,30]. There is also a possibility that environmental activities changed with certain seasons, such as farming behaviors. There is also potential for information bias due to possible human error in the sampling protocols or laboratory analyses. With thousands of public water systems taking samples, it is unknown if the people taking samples were following correct protocol in taking the sample and packaging it to send to the EPA-designated laboratories for analysis, and there is the possibility that equipment was not calibrated properly for each sample that was taken [29,30].

There is suggestive evidence for potential toxicological interaction between chromium and vanadium. A cross-sectional analysis of data from a Canadian cohort of 36 children and adolescents with chronic kidney disease [31] found that these patients had elevated levels of vanadium and chromium in their blood plasma. This may be influenced by a low estimated glomerular filtration rate as well as environmental exposure. A toxicology study of 48 Wistar rats found that rats that were co-exposed to water solutions of vanadium and chromium for 12 weeks had significantly higher levels of Malondialdehyde (MDA) in the liver than the control group, which is a biomarker of oxidative stress [32]. A randomized experiment examined the red blood cells of 56 Wistar rats divided into four groups that received doses of vanadium, chromium, and a chromium/vanadium solution. The study found that the rats that were co-exposed to vanadium and chromium for 12 weeks had a significant decrease in glutathione s-transferase activity (GST) [33]. These results are preliminary; however, they suggest the possibility that a co-exposure may inhibit GST activity. 

## 5. Conclusions

The co-occurrence of vanadium and chromium in public water systems, especially in small water systems, raises the possibility of human joint exposures to these drinking water contaminants. Additional research is needed to inform cumulative risk assessment for these contaminants.

## Figures and Tables

**Table 1:** The number of all water systems that reported all concentration values below the MRL (<) is listed for each of the six metals.

Contaminants	<	Total	%
Chromium	1262	4922	25.64%
Molybdenum	2375	4922	48.25%
Hexavalent Chromium	518	4919	10.53%
Cobalt	4675	4922	94.98%
Vanadium	1419	4761	29.80%
Strontium	0	4922	0.00%

**Table 2:** Pairwise correlations of predicted geometric means of large public water systems (>10,000 people served) by contaminant with *p* value for the correlation on the second line.

Variables	Cr	Sr	Cr (VI)	Mo	Co	V
Cr	1					
Sr	0.036 *	1				
	<0.01					
Cr (VI)	0.984 *	0.032 *	1			
	<0.01	<0.01				
Mo	0.008	0.097 *	0.01	1		
	0.1	<0.01	0.05			
Co	−0.017 *	−0.024 *	−0.021 *	−0.003	1	
	<0.01	<0.01	<0.01	0.53		
V	0.448 *	0.011	0.445 *	0.045 *	−0.046 *	1
	<0.01	0.02	<0.01	<0.01	<0.01	

* Bonferroni-significant correlation.

**Table 3:** Pairwise correlations of predicted geometric means of small public water systems (≤10,000 people served) by contaminant with *p* value for the correlation on the second line.

Variables	Cr	Sr	Cr (VI)	Mo	Co	V
Cr	1					
Sr	0.074 *	1				
	<0.01					
Cr (VI)	0.960 *	0.069 *	1			
	<0.01	<0.01				
Mo	0.042 *	0.138 *	0.046 *	1		
	<0.01	<0.01	<0.01			
Co	−0.034 *	−0.027 *	−0.040 *	0.023 *	1	
	<0.01	<0.01	<0.01	<0.01		
V	0.474 *	0.066 *	0.498 *	0.080 *	−0.063 *	1
	<0.01	<0.01	<0.01	<0.01	<0.01	

* Bonferroni-significant correlation.

## Data Availability

The publicly available dataset that informed this study is available from the Environmental Protection Agency here: https://www.epa.gov/dwucmr/occurrence-data-unregulated-contaminant-monitoring-rule#3. (Accessed on 14 February 2019).
